# Редкий случай кортикотропин-продуцирующей феохромоцитомы в составе синдрома множественных эндокринных неоплазий 1 типа

**DOI:** 10.14341/probl13260

**Published:** 2023-11-11

**Authors:** Д. В. Реброва, С. И. Григорова, Н. В. Ворохобина, Е. А. Згода, К. Ю. Новокшонов, С. Г. Феофанова, В. Ф. Русаков, Л. М. Краснов, Е. А. Федоров, И. К. Чинчук, Ш. Ш. Шихмагомедов, А. А. Пушкарук, И. В. Слепцов

**Affiliations:** Санкт-Петербургский государственный университет, Клиника высоких медицинских технологий им. Н. И. Пирогова; Ленинградская областная клиническая больница; Северо-Западный государственный медицинский университет им. И.И. Мечникова; Санкт-Петербургский государственный университет, Клиника высоких медицинских технологий им. Н.И. Пирогова; Санкт-Петербургский государственный университет, Клиника высоких медицинских технологий им. Н.И. Пирогова; Ленинградская областная клиническая больница; Санкт-Петербургский государственный университет, Клиника высоких медицинских технологий им. Н.И. Пирогова; Санкт-Петербургский государственный университет, Клиника высоких медицинских технологий им. Н.И. Пирогова; Санкт-Петербургский государственный университет, Клиника высоких медицинских технологий им. Н.И. Пирогова; Санкт-Петербургский государственный университет, Клиника высоких медицинских технологий им. Н.И. Пирогова; Санкт-Петербургский государственный университет, Клиника высоких медицинских технологий им. Н.И. Пирогова; Санкт-Петербургский государственный университет, Клиника высоких медицинских технологий им. Н.И. Пирогова; Санкт-Петербургский государственный университет, Клиника высоких медицинских технологий им. Н.И. Пирогова

**Keywords:** феохромоцитома, синдром множественных эндокринных неоплазий 1 типа, первичный гиперпаратиреоз, синдром Кушинга, эктопическая секреция АКТГ, кортикотропин

## Abstract

В статье представлен клинический случай мужчины 66 лет, у которого была выявлена гормонально-неактивная макроаденома гипофиза, осложненная эрозией роговицы и частичной атрофией зрительного нерва левого глаза вследствие экзофтальма. Повышение уровня пролактина было расценено как «stalk-effect». Пациенту была выполнена трансназальная аденомэктомия гипофиза с последующим регрессом симптомов. Через 4 года на фоне новой коронавирусной инфекции появились нарастающая общая слабость, головные боли, кризовое повышение уровня артериального давления и приступы тахикардии. При компьютерной томографии (КТ) случайно выявлено новообразование надпочечника, лабораторно — выраженный гиперкортизолизм, повышенный уровень адренокортикотропного гормона (АКТГ), гипокалиемия, гипергликемия, повышение уровней метанефрина и норметанефрина. В стационаре у пациента развился острый стероидный психоз, после купирования которого выполнена адреналэктомия с опухолью, гистологически подтверждена феохромоцитома. После операции отмечались регресс симптоматики, развитие надпочечниковой недостаточности с пониженными уровнями АКТГ и кортизола. При дальнейшем обследовании установлен полинодозный эутиреоидный зоб, по данным тонкоигольной аспирационной биопсии узлов — тиреоидит Хашимото (Bethesda II). Лабораторно выявлен первичный гиперпаратиреоз. По данным УЗИ, сцинтиграфии с Тс99m-Технетрилом и КТ выявлено увеличение околощитовидной железы (ОЩЖ) слева. Выполнены двусторонняя ревизия шеи, удаление аденом правой верхней и левой верхней ОЩЖ. В послеоперационном периоде нормализовались уровни кальция и паратгормона. Учитывая наличие сочетания множественных опухолей эндокринной системы (первичного гиперпаратиреоза, кортикотропин-продуцирующей феохромоцитомы, гормонально-неактивной макроаденомы гипофиза, многоузлового нетоксического зоба), установлен синдром множественных эндокринных неоплазий 1 типа (МЭН1) клинически. При исследовании 2 и 10 экзонов гена MEN1 мутаций не выявлено, что не исключает наличие наследственного синдрома. Больной находится под наблюдением. Пациентов с эктопической гиперпродукцией АКТГ феохромоцитомой в составе синдрома МЭН1, подобных описанному в данной статье, в доступной литературе на русском и английском языках найдено не было, в связи с чем есть основания считать представленный случай первым.

## Abstract (en)

A clinical case of a man 66 y.o. who was diagnosed with hormone-inactive pituitary macroadenoma complicated by corneal erosion and partial atrophy of the optic nerve of the left eye due to exophthalmos. The increase in prolactin level was regarded due to a «stalk-effect». The patient underwent a transnasal pituitary adenomectomy with subsequent regression of symptoms. After 4 years, against the background of a new coronavirus infection, increasing general weakness, headaches, a crisis increase in blood pressure and tachycardia attacks appeared. Computed tomography (CT) accidentally revealed an adrenal incidentaloma, in laboratory tests — hypercortisolism, elevated ACTH levels, hypokalemia, hyperglycemia, increased levels of metanephrine and normetanephrine. The patient developed acute steroid psychosis, after which an adrenalectomy with a tumor was performed, a pheochromocytoma was histologically confirmed. After surgery, there was a regression of symptoms, the development of adrenal insufficiency with reduced levels of ACTH and cortisol. Upon further examination, a polynodose euthyroid goiter was established, the biopsy of the nodes — Hashimoto’s thyroiditis (Bethesda II). Meanwhile, primary hyperparathyroidism was detected. According to ultrasound, scintigraphy with Ts99m-Technetril and CT revealed an increase of left parathyroid gland. A bilateral revision of the neck, removal of the right upper and left upper parathyroid adenomas were performed. In the postoperative period, the levels of calcium and parathyroid hormone were normalized. Given the presence of a combination of multiple tumors of the endocrine system (primary hyperparathyroidism, corticotropin-producing pheochromocytoma, hormone-inactive pituitary macroadenoma, polynodose euthyroid goiter), the MEN1 syndrome was clinically established. The study of 2 and 10 exons of the MEN1 gene revealed no mutations, which does not exclude the presence of a hereditary syndrome. The patient continues observation. In the available literature in Russian and English languages the case of ACTH pheochromocytoma as part of the MEN type 1 syndrome have not been found. Therefore, we consider the presented case to be the first one.

## АКТУАЛЬНОСТЬ

Множественные эндокринные неоплазии (МЭН) — редкие генетические синдромы с аутосомно-доминантным типом наследования, отличающиеся высокой пенетрантностью. Для каждого синдрома характерно развитие опухолей или гиперплазии нескольких эндокринных органов. В настоящее время выделяют 4 типа синдромов МЭН.

Для синдрома МЭН 1 типа (синдром Вермера) наиболее характерно развитие первичного гиперпаратиреоза с множественным поражением околощитовидных желез с пенетрантностью более 90–95% [1]. У 30–70% пациентов развиваются нейроэндокринные опухоли (НЭО), из них в 80% случаев процесс локализуется в поджелудочной железе и двенадцатиперстной кишке (гастринома, инсулинома, PP-ома, гормонально-неактивные опухоли) [3]. Реже встречаются НЭО желудка, кишечника, тимуса и легких. Очень редко (менее 1% случаев) глюкагонома и опухоль, продуцирующая вазоактивный интестинальный пептид (ВИПома) [1][3]. В 30–40% случаев обнаруживают опухоли гипофиза (пролактинома, соматотропинома, кортикотропинома, гормонально-неактивные аденомы), в 40% случаев — доброкачественные новообразования надпочечников, которые могут быть как с гормональной активностью, так и без нее [3]. Адренокортикальный рак и феохромоцитома встречаются очень редко (менее 1% случаев) [1]. Кроме того, у 40% пациентов с МЭН 1 типа могут быть неэндокринные проявления: различные кожные образования (подкожные липомы, ангиофибромы и коллагеномы), опухоли центральной нервной системы (менингиомы, эпендимомы, шванномы) и гладкой мускулатуры (лейомиомы). Кроме того, у женщин с данным наследственным синдромом обнаруживается повышенный риск рака молочной железы. Кожные изменения могут предшествовать развитию клинической картины [2]. Появление синдрома МЭН 1 типа ассоциировано с мутацией гена MEN1. На настоящий момент известно около 1200 разных мутаций данного гена, но в 5–25% случаев обнаружить их доступными методами не удается [3].

Фенотипически схожим с синдромом МЭН 1 типа является выявленный в последнее десятилетие синдром МЭН 4 типа, который ассоциирован с инактивирующей мутацией гена супрессора опухоли CDKN1B. Считается, что около 1–2% семей с клинически установленным синдромом МЭН 1 типа на самом деле имеют МЭН 4 типа [2]. Для пациентов с синдромом МЭН 4 типа характерно сочетание первичного гиперпаратиреоза, опухолей гипофиза, надпочечников, почек, поджелудочной железы, карциноидов бронхов и желудка, гастриномы, рака молочной железы, шейки матки, яичка, а также встречается папиллярная карцинома щитовидной железы [4].

При синдромах МЭН 2 типа практически в 100% случаев встречается медуллярная карцинома щитовидной железы, в 50–75% случаев диагностируют феохромоцитому. Для синдрома МЭН 2А типа (синдром Сиппла) характерно развитие первичного гиперпаратиреоза, болезни Гиршпрунга (аганглиоз толстой кишки), кожного лихеноидного амилоидоза. При синдроме МЭН 2В типа (синдром Горлина) у пациентов выявляется марфаноподобная внешность, деформация грудной клетки и стоп, а также ганглионевромы конъюнктивы глаз, роговичного нерва, слизистых ЖКТ (языка, рта, кишечника) [5][6].

Диагностика синдромов МЭН основана на выявлении клинических проявлений при сочетании двух или более характерных для синдрома опухолей. Также диагноз может быть поставлен на основе обнаружения одной опухоли при наличии родственника первой степени родства с установленным соответствующим синдромом или при подтверждении ассоциированной генетической мутации даже в отсутствие признаков заболевания [3][6].

В настоящей статье представлено описание редкого случая кортикотропин-продуцирующей феохромоцитомы при синдроме МЭН.

## ОПИСАНИЕ СЛУЧАЯ

Пациент К., 66 лет, поступил в эндокринологическое отделение ГБУЗ «Ленинградская областная клиническая больница» (ЛОКБ) 21.03.2021 с жалобами на нарастающую общую слабость, быструю утомляемость, выраженную слабость в ногах, головокружение, шум в ушах, дрожь в руках, сильную потливость, изжогу, одышку при незначительной физической нагрузке, повышение уровня артериального давления (АД) максимально до 270 и 140 мм рт. ст., запоры до 3–5 сут.

В течение последнего года пациента беспокоили осиплость и огрубение голоса, выраженные отеки поясницы, кистей, лица, нижних конечностей до уровня коленных суставов. За последние полгода было отмечено снижение массы тела на 10 кг.

Из анамнеза известно, что ухудшение состояния пациент стал отмечать с 2014 г., когда появились жалобы на головные боли, головокружения, нарушения зрения и слуха, ухудшение памяти. В 2016 г. в связи с присоединением чувства давления в области глазниц и появлением одностороннего экзофтальма был направлен на визуализирующее исследование.

По данным магнитно-резонансной томографии (МРТ) от 21.04.2016 в хиазмально-селлярной области (ХСО) определено образование размерами 26×25×25 мм с однородной структурой, прилежащее и оттесняющее кверху перекрест зрительных нервов, интимно прилежащее к внутренним сонным артериям, сжимающее прилежащие отделы в проекции прямой извилины левой лобной доли, супраселлярную цистерну и передние отделы III желудочка. Отмечено пролабирование дна турецкого седла в клиновидную пазуху.

По результатам гормонального обследования данных за гормональную активность получено не было. Кортикотропин (АКТГ) составил 2,881 пмоль/л (1,034–10,736), фолликулостимулирующий гормон (ФСГ) — 1,46 мМЕ/мл (1,27–19,26), лютеинизирующий гормон (ЛГ) — 1,58 мМЕ/мл (1,24–8,62), соматотропный гормон (СТГ) — 0,311 нг/мл (0,003–0,971), тиреотропный гормон (ТТГ) — 6,0742 мкМЕ/мл (0,35–4,94). Полученное повышение уровня пролактина до 1277,87 мМЕ/мл (55,97–278,36) было трактовано как «stalk-effect» (эффект сдавленной ножки гипофиза с нарушением транспорта дофамина и, соответственно, увеличением секреции пролактина). Офтальмологом были выявлены эрозия роговицы и частичная атрофия зрительного нерва левого глаза вследствие экзофтальма.

В 2016 г. пациенту была выполнена трансназальная аденомэктомия гипофиза. По данным контрольной МРТ после оперативного вмешательства визуализированы кистозно-фиброзные изменения в области дна турецкого седла и остаточная ткань образования вдоль переднелевой стенки турецкого седла, пролабирование супраселлярной цистерны в полость турецкого седла, смещение воронки гипофиза вправо. Далее вплоть до 2021 г. контроль инструментальных и гормональных исследований не проводился.

С 2019 г. у пациента стало повышаться АД до 170–180 и 90–100 мм рт. ст., в связи с чем назначена гипотензивная терапия (лозартан), что способствовало значительному улучшению самочувствия.

Ухудшение состояния отмечено с середины 2020 г., когда у пациента появились сильные головные боли, повышенная потливость, нарастающая общая слабость. В январе 2021 г. на фоне новой коронавирусной инфекции периодически уровень АД повышался до 270 и 140 мм рт. ст., что сопровождалось ощущением дрожи рук и тела, чувством «жара», повышенной потливостью. В связи с ухудшением состояния в феврале 2021 г. госпитализирован в городскую больницу города Санкт-Петербурга.

Учитывая выявленные участки гипокинезии миокарда по результатам эхокардиографии, пациенту была выполнена коронарография. Визуализирован стеноз 20–30% дистальной трети левой огибающей артерии (LCx) с удовлетворительным периферическим кровотоком.

При выполнении компьютерной томографии (КТ) органов грудной клетки обнаружено новообразование медиальной ножки левого надпочечника размерами 33×35×35 мм с нативной плотностью +22 HU, неоднородной структуры. Кортизол в сыворотке крови утром был 1299 нмоль/л (154–657), вечером — 1044 нмоль/л, уровень калия в крови колебался от 2,7 до 3,2 ммоль/л (3,5–5,1), содержание глюкозы в крови повышалось до 12,1 ммоль/л (4,1–6,0). После «ночного» теста с 1 мг дексаметазона кортизол сыворотки крови составил 1044,9 нмоль/л (подавления выработки кортизола не было). По клиническим и лабораторным данным у пациента заподозрен эндогенный гиперкортицизм.

На фоне приема спиронолактона (200 мг в сутки), бисопролола (2,5 мг в сутки), нифедипина (10 мг в сутки), торасемида (10 мг в сутки), препаратов калия сохранялось приступообразное повышение уровня АД (до 180 и 100 мм рт. ст.), сопровождающееся чувством «жара в теле», дрожью в руках, повышенной потливостью, чувством страха, общей слабостью. Приступы беспокоили чаще в ранние утренние часы, купировались самостоятельно. Уровень АД 160 и 90 мм рт. ст. пациент субъективно не ощущал. Уровень гликемии натощак снизился без назначения терапии до 6,6 ммоль/л. После выписки пациент самостоятельно снизил дозу спиронолактона до 100 мг в сутки с последующим прекращением приема препарата.

В марте 2021 г. пациент госпитализирован в ЛОКБ с целью уточнения гормональной активности новообразования надпочечника. При поступлении в стационар отмечались небольшая осиплость голоса, никтурия, гиперпигментация и сухость кожных покровов, умеренная инъекция склер, гипотрофия мышц конечностей, тремор кистей рук, отеки лица, кистей рук, нижних конечностей до уровня середины бедра. Уровень АД составил 180 и 100 мм рт. ст., стул, со слов пациента, был 1 раз в 3–5 дней, оформленный (с использованием очистительных клизм).

По данным лабораторного исследования обращали на себя внимание лейкоцитоз до 11,14×109/л в клиническом анализе крови (миелоциты 2%, палочкоядерные 5%, сегментоядерные 76%), повышение уровня С-реактивного белка до 9,06 мг/л (0–5), гипопротеинемия (общий белок 52,7 г/л при норме 60–80), дислипидемия (общий холестерин 7,5 ммоль/л, липопротеиды низкой плотности 4,11 ммоль/л, триглицериды 2,25 ммоль/л), гипергликемия до 10–15 ммоль/л, гликированный гемоглобин 10%, гипокалиемия (2,5–2,7 ммоль/л при норме 3,5–5,5), гиперкоагуляция (активированное парциальное тромбопластиновое время 17,2 с при норме 23,0–31,9, фибриноген 4,31 г/л при норме 1,80–3,50).

При гормональном обследовании в крови АКТГ в 08:00 был 158 пг/мл (до 46), в 23:00 — 116 пг/мл, кортизол в 8:00 — 952 нмоль/л (138–690), в 23:00 — 811 нмоль/л, дегидроэпиандростерон-сульфат — 64 мкг/дл (80–560), альдостерон — 113,9 пг/мл (до 199), ренин — менее 2,0 пг/мл (2,13–58,78), метанефрин — 95,2 пг/мл (до 65), норметанефрин — 4675,7 пг/мл (до 196), пролактин — 104,0 мМЕ/мл (53–360), инсулиноподобный фактор роста 1 (ИФР-1) — 168,5 нг/мл (37,0–236,0), ТТГ — 0,3 мкМЕ/мл (0,4–4,0), свободный тироксин — 10,9 пмоль/л (9,0–19,5), антитела (АТ) к тиреопероксидазе — 0,39 МЕ/мл (менее 5,6), АТ к рецепторам ТТГ — 0,21 МЕ/мл (менее 1,8), кальцитонин — 3,6 пг/мл (до 14,3), паратгормон — 153,3 пг/мл (15–65), кальций общий — 2,42 ммоль/л (2,15–2,50), фосфор — 0,9 ммоль/л (0,7–1,6), кальций ионизированный — 1,21 ммоль/л (1,12–1,29). После ночного подавляющего теста с 1 мг дексаметазона уровень кортизола составил 1380 нмоль/л (подавление выработки кортизола не достигнуто).

По результатам проведения МРТ ХСО с контрастированием турецкое седло расширено до 10,7×17,4×12,3 мм, анте- и параселлярно слева определен участок с изоинтенсивным магнитно-резонансным (МР) сигналом размерами 6,6,×6,7×10 мм с четкими ровными контурами, воронка гипофиза расположена справа от срединной линии, хиазма и кавернозные синусы интактны. На серии постконтрастных МР-томограмм отмечено неинтенсивное накопление контрастного вещества обнаруженным участком анте- и параселлярно слева (рис. 1).

**Figure:**
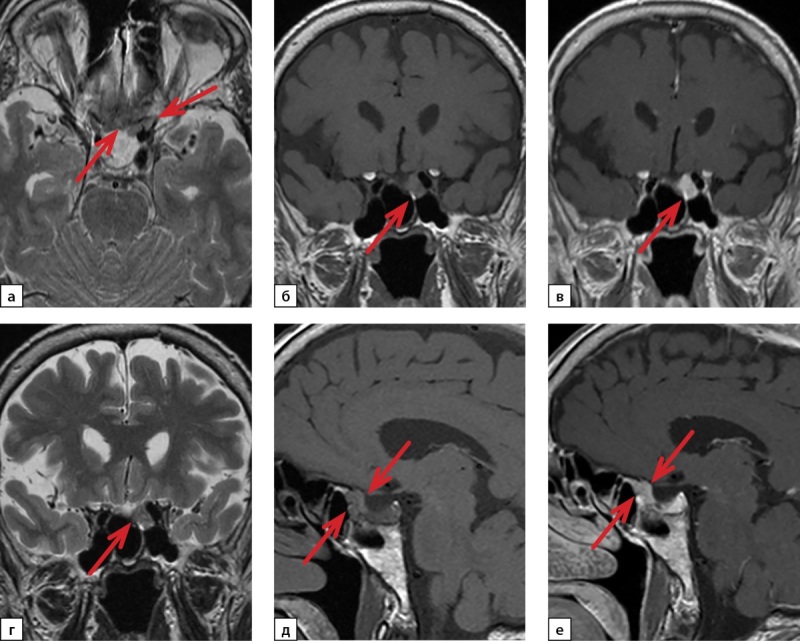
Рисунок 1. Магнитно-резонансная томография хиазмально-селлярной области с контрастированием: а, г — Т2-ВИ, б, д — Т1-ВИ, в, е — Т1-ВИ с контрастированием.

По данным УЗИ общий объем щитовидной железы не увеличен, структура диффузно неоднородная, в нижней трети правой доли лоцируется неоднородный изоэхогенный узел с ободком halo размерами 14×11 мм, в средней трети обнаружены два гипоэхогенных узла размерами 6×4 мм и 5×4 мм, в верхней трети — аналогичный узел размерами 4×2 мм, на границе с перешейком — изоэхогенный узел с ободком halo размерами 10×6 мм. В нижней трети левой доли по переднему контуру определен изоэхогенный узел с нечетким гипоэхогенным ободком размерами 7×5 мм.

По результатам УЗИ органов брюшной полости и почек выявлены гепатомегалия, повышение эхогенности печени, диффузные изменения поджелудочной железы. Патологии почек и надпочечников не обнаружено.

В результате проведения КТ брюшной полости и забрюшинного пространства с контрастированием (рис. 2) в левом надпочечнике визуализировано округлое образование размерами 30×33 мм, плотностью +16–(+)30 HU, с ровными и четкими контурами, неравномерно накапливающее контрастный препарат (в портальную фазу контрастирования до +83 HU, в отсроченную фазу — без значимого вымывания контрастного препарата; в структуре определены участки, не накапливающие контрастный препарат).

**Figure:**
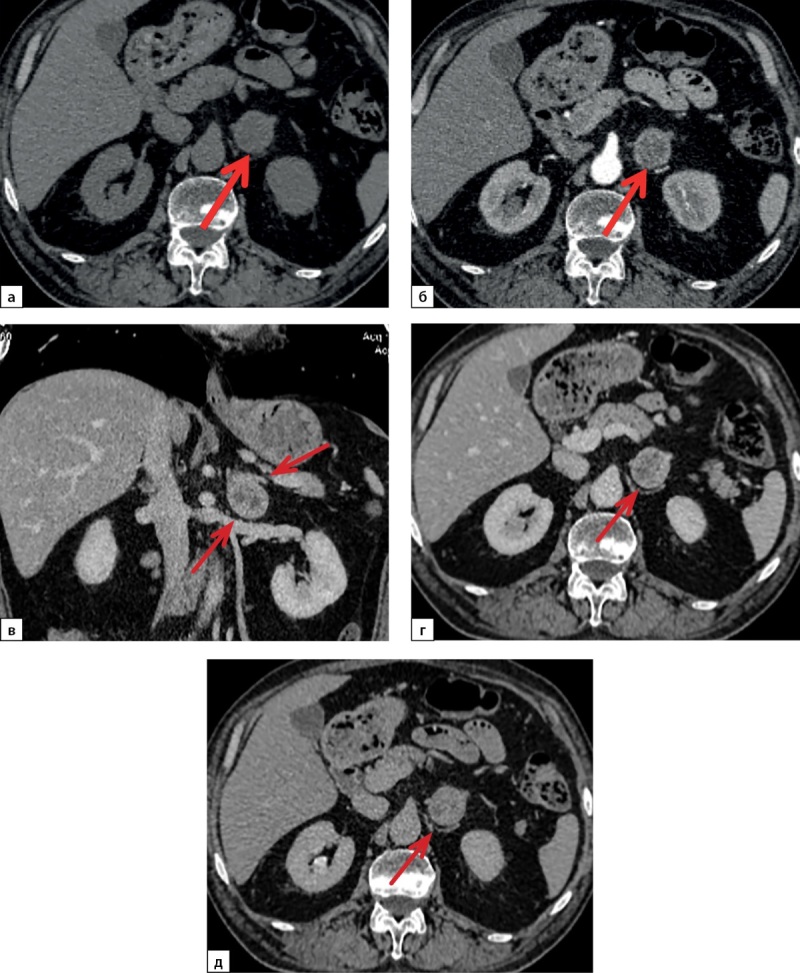
Рисунок 2. Компьютерная томография органов брюшной полости с контрастированием: а — нативное сканирование; б — артериальная фаза; в, г — венозная фаза; д — отсроченная фаза.

По заключению рентгенографии грудного и поясничного-крестцового отделов позвоночника данных за компрессионные переломы не получено.

Электрокардиографическое исследование выявило отклонение электрической оси сердца влево, выраженную гипертрофию левого желудочка, гипертрофию левого предсердия, нарушения процессов реполяризации.

На момент госпитализации кризы с повышением уровня АД, сопровождающиеся симпатоадреналовой симптоматикой, наблюдались 1 раз в 1–2 дня. С целью уменьшения количества катехоламиновых кризов в стационаре проводилась терапия доксазозином с постепенной титрацией дозы до 16 мг в сутки, амлодипином в дозе 10 мг в сутки, лозартаном 100 мг в сутки, бисопрололом 2,5 мг в сутки. На фоне терапии кризы стали реже — 1 раз в 3–5 дней, с повышением уровня АД не выше 200 и 120 мм рт. ст., учащением пульса не более 85 в минуту. Для коррекции гипокалиемии назначены лечение спиронолактоном с постепенной титрацией дозировки до 400 мг в сутки, внутривенные инфузии и пероральный прием препаратов калия. Для профилактики тромбозов после перенесенной новой коронавирусной инфекции пациент получал апиксабан 2,5 мг 2 раза в день, в связи с массивными отеками — фуросемид 80 мг в сутки, с целью коррекции гипергликемии — инсулинотерапию в базис-болюсном режиме.

С целью дифференциальной диагностики гиперпаратиреоза на фоне нормального уровня кальция крови выполнен тест с альфакальцидолом (прием в дозе 1 мкг в сутки в течение 7 дней). В результате теста определена гиперкальциемия — общий кальций 2,72 ммоль/л (2,15–2,50), патологическое повышение уровня паратгормона до 177,8 пг/мл (15–65), что подтвердило наличие первичного гиперпаратиреоза.

С учетом тяжести состояния больного, невозможности проведения селективного забора крови из каменистых синусов для определения уровня АКТГ, с целью исключения эктопической продукции кортикотропина планировалось проведение позитронно-эмиссионной томографии, совмещенной с КТ (ПЭТ/КТ) всего тела с 18-фтордезоксиглюкозой.

При обследовании через неделю после госпитализации выявлено нарастание содержания в крови уровней АКТГ до 293 пг/мл (менее 46), кортизола более 1380 нмоль/л (138 –690).

09.04.2021 во второй половине дня у пациента (АД 135/80 мм рт. ст., гликемия 9,0 ммоль/л) возникло состояние острого соматогенного стероидного психоза с выраженным психомоторным возбуждением, агрессией, зрительными и слуховыми галлюцинациями, потребовавшее вызова невролога. Острое нарушение мозгового кровообращения было исключено по данным КТ головного мозга. В связи с неадекватным агрессивным поведением в течение 2 сут пациент был фиксирован, получал седативную терапию, на фоне чего состояние стабилизировалось, галлюцинации регрессировали. Учитывая прогрессирование проявлений гиперкортицизма, консилиумом было принято решение о проведении левосторонней адреналэктомии без дополнительного обследования.

22.04.2021 выполнена ретроперитонеоскопическая левосторонняя адреналэктомия. Получено описание послеоперационного макропрепарата. В макропрепарате определены фрагмент жировой клетчатки размерами 60×35×30 мм, структура надпочечника на разрезе размерами 45×28×28 мм с образованием в мозговом веществе 26 мм в диаметре (с четкой границей, с кровоизлияниями). В ткани надпочечника без связи с образованием выявлен округлый узел светло-желтого цвета диаметром 6 мм с четкими границами. В гистологическом заключении указано на наличие феохромоцитомы диаметром 26 мм и светлоклеточной адренокортикальной аденомы диаметром 6 мм надпочечника по морфологическим признакам. Результаты иммуногистохимического исследования: клетки образования диффузно ярко экспрессируют Chromatogranin A, Synaptophisin, S100, отсутствует экспрессия СК АЕ1/АE3, Melan A, что подтверждает наличие феохромоцитомы; индекс пролиферативной активности Ki-67 менее 1%.

Интраоперационно сразу после адреналэктомии у пациента развился эпизод гипотонии до 80 и 50 мм рт.ст., который был купирован введением препаратов глюкокортикоидов и норадреналина. На 2-е сутки после операции в связи с развитием надпочечниковой недостаточности (АКТГ менее 5 пг/мл, кортизол крови 45 нмоль/л) назначена заместительная гормональная терапия глюкокортикоидами. Через сутки после удаления опухоли надпочечника в плазме метанефрин в крови — 8,4 пг/мл (до 65), норметанефрин — 228,3 пг/мл (до 196).

В послеоперационном периоде клинически отмечались регресс эмоциональной лабильности, агрессии, общей и мышечной слабости, посветление кожных покровов, стабилизация показателей гемодинамики (уровень АД 120–130 и 80 мм рт.ст.) на фоне получаемой заместительной гормональной терапии, нормокалиемия на фоне отмены спиронолактона и препаратов калия, нормогликемия без сахароснижающей терапии. У пациента сохранялись умеренные отеки нижних конечностей, что потребовало возобновления диуретической терапии (торасемид 10 мг и спиронолактон 25 мг в сутки) с положительной динамикой.

Пациент был выписан на амбулаторное лечение с рекомендациями для обследования по поводу многоузлового зоба и первичного гиперпаратиреоза для определения тактики лечения. На момент выписки из стационара пациент получал кортизон ацетат 50 мг в сутки с последующим снижением дозы амбулаторно до 25 мг в сутки.

Амбулаторно выполнена сцинтиграфия щитовидной и околощитовидных желез 01.07.2021:

10.06.2021 проведена денситометрия, подтвердившая наличие остеопороза. В поясничных позвонках L1–L4 показатели минеральной плотности костной ткани были низкими: Т-критерий в L1–L4 составил -2,7 SD, что соответствует снижению костной массы от пиковой для возраста минимально на 25% (в L2) и максимально на 31% (в L4). В проксимальных отделах левой бедренной кости показатели минеральной плотности костной ткани также были снижены (Т-критерий в шейке бедренной кости до -2,3 SD, в проксимальных отделах бедренной кости до -2,5 SD, что соответствует снижению костной массы от пиковой для возраста на 27 и 32% соответственно).

В сентябре 2021 г. пациент госпитализирован в Клинику высоких медицинских технологий им. Н.И. Пирогова (КВМТ) СПбГУ.

По данным КТ шеи с внутривенным контрастированием щитовидная железа расположена обычно, в размерах не увеличена. Вдоль задней поверхности среднего и нижнего отделов правой доли щитовидной железы визуализировано образование размерами 9×5×13 мм, гиподенсное по отношению к ткани щитовидной железы при нативном исследовании, интенсивно накапливающее контрастное вещество в артериальную фазу, «сбрасывающее» контраст в венозную фазу исследования. Определены множественные участки разряжения костной структуры до 9 мм в костях черепа и телах позвонков в зоне сканирования, более вероятно, обусловленные гиперпаратиреоидной остеодистрофией. Справа вдоль яремной вены — лимфатические узлы до 0,5 см в диаметре, слева до 0,6 см в диаметре.

Выполнена тонкоигольная аспирационная биопсия (ТАБ) узлов щитовидной железы: в изоэхогенном узле в центре правой доли (EU-TIRADS 4) и изоэхогенном узле на границе с перешейком (EU-TIRADS 4) установлена цитологическая картина тиреоидита Хашимото (Bethesda II). Также выполнена ТАБ лимфатического узла 9 мм в среднеяремной зоне справа, при цитологическом исследовании которого обнаружено обилие клеточных элементов лимфоидной ткани без признаков опухолевого поражения.

Перед операцией кальций общий в крови был 2,69 ммоль/л (2,15–2,55), кальций ионизированный — 1,36 ммоль/л (1,13–1,31), 25ОН-витамин D — 14,61 нг/мл (30–100), паратгормон — 9,9 пмоль/л (1,3–9,3).

Электрокардиограмма и эхокардиограмма — без существенной патологии.

20.09.2021 выполнена двусторонняя ревизия шеи, удаление аденом правой и левой верхних околощитовидных желез. По результатам гистологического заключения послеоперационного материала удалены аденома (1,2 см) правой верхней околощитовидной железы и левая верхняя околощитовидная железа (1,1 см) с диффузным липоматозом. В послеоперационном периоде в крови нормализовались уровни кальция ионизированного — 1,17 ммоль/л (1,13–1,31) и паратгормона — 4,4 пмоль/л (1,3–9,3) (табл. 1).

**Table:** Таблица 1. Динамика лабораторных показателей пациента К.

Показатель	До операции на гипофизе	До операции на надпочечнике	Тест с альфакальцидолом	До операции на околощитовидных железах	После операции на околощитовидных железах	Референсные значения
АКТГ, пмоль/л	2,881					1,034–10,736
АКТГ, пг/мл		239				до 46
Кортизол, нмоль/л		>1380				138–620
Ночной тест с 1 мг дексаметазона–кортизол, нмоль/л		1044,9				до 50
ФСГ, мМЕ/мл	1,46					1,27– 19,26
ЛГ, мМЕ/мл	1,58					1,24–8,62
СТГ, нг/мл	0,311					0,003–0,971
Инсулиноподобный фактор роста 1 (ИФР-1), нг/мл		168,5				37,0–236,0
Тиреотропный гормон (ТТГ), мкМЕ/мл	6,0742	0,3				0,4–4,0
Свободный тироксин (Т4), пмоль/л		10,9				9,0–19,5
Антитела (АТ) к тиреопероксидазе (ТПО), МЕ/мл		0,39				менее 5,6
АТ к рецепторам ТТГ, МЕ/мл		0,21				менее 1,8
Кальцитонин, пг/мл		3,6				до 14,3
Пролактин, мМЕ/мл	1277,87	104				53–360
Дэгидроэпиандростерон-сульфат (ДЭА-с), мкг/дл		64				80–560
Альдостерон, пг/мл		113,9				до 199
Ренин, пг/мл		менее 2,0				2,13–58,78
Метанефрин, пг/мл		95,2	8,4			до 65
Норметанефрин, пг/мл		4675,7	228,3			до 196
Паратгормон, пг/мл		153,3	177,8			15–65
Паратгормон, пмоль/л				9,9	4,4	1,3–9,3
Калий, ммоль/л		2,5				3,5–5,1
Кальций общий, ммоль/л		2,42	2,72	2,69		2,15–2,50
Кальций ионизированный, ммоль/л		1,21		1,36	1,17	1,13–1,31
Фосфор, ммоль/л		0,9				0,7–1,6
25ОН-витамин D, нг/мл				14,61		30–100
Глюкоза, ммоль/л		12,1		5,7		
Гликированный гемоглобин, %		10				

Пациенту был рекомендован прием альфакальцидола в дозе 1 мкг в сутки, карбоната кальция по 500 мг 2 раза в день.

Учитывая наличие сочетания множественных опухолей эндокринной системы (первичного гиперпаратиреоза, кортикотропин-продуцирующей феохромоцитомы, гормонально-неактивной макроаденомы гипофиза, многоузлового нетоксического зоба), было выполнено генетическое исследование: патогенных вариантов и мутаций в экзоне 10,11,13–16 гена RET и экзонах 2,10 гена MEN1 обнаружено не было.

## ОБСУЖДЕНИЕ

Феохромоцитома (ФЕО) и параганглиома (ПГ) — опухоли хромаффинной ткани, продуцирующие катехоламины (адреналин, норадреналин, дофамин). В связи с нейроэндокринным происхождением данных неоплазий описаны крайне редкие случаи эктопической секреции и других пептидных гормонов, таких как вазоактивный интестинальный пептид, соматостатин, нейропептид Y, метэнкефалин, кальцитонин, АКТГ [7– 9].

Редкость смешанной гормональной продукции одной опухолью представляет трудность в дифференциальной диагностике подобных случаев с наличием сочетанной патологии с гиперсекрецией гормонов из разных источников. В представленном клиническом случае наличие смешанной артериальной гипертензии с пароксизмальными приступами повышения уровня АД, сопровождающейся ощущением дрожи в теле, тремором рук, выраженной общей слабостью, повышенной потливостью, чувством страха, в сочетании с выявленными повышенными уровнями метанефрина и норметанефрина плазмы свидетельствовали в пользу катехоламин-продуцирующей опухоли. Обращал на себя внимание факт незначительного повышения уровня метанефрина при превышении уровня норметанефрина более чем на 20 норм, что не позволяло исключить наличие ПГ. При КТ визуализировано новообразование надпочечника высокой плотности, однако распределение контрастного вещества с низким накоплением в артериальную фазу, максимальным накоплением в венозную фазу и слабым выведением в отсроченную фазу, что более характерно для адренокортикальных карцином, чем для ФЕО [10–12].

Трудно корригируемая артериальная гипертензия в сочетании с сахарным диабетом, гипокалиемия с выраженной общей и мышечной слабостью, гиперкоагуляция, гипопротеинемия, данные объективного исследования, развитие психоэмоциональных расстройств, высокие уровни АКТГ и кортизола в утренние и вечерние часы, отсутствие подавления уровня кортизола после приема дексаметазона свидетельствовали в пользу АКТГ-зависимого синдрома Кушинга. Тенденция к сохранению суточного ритма секреции АКТГ и кортизола, наличие макроаденомы гипофиза с частичным удалением последней в анамнезе не позволяли исключить гипофизарную форму гиперкортицизма. Снижение уровня ТТГ было расценено как вторичный гипотиреоз на фоне гиперкортизолемии. Селективный забор крови из каменистых синусов не был выполнен в связи с тяжестью состояния больного.

Топическая диагностика ФЕО или ПГ, НЭО с эктопической продукцией АКТГ могла быть дополнена ПЭТ/КТ всего тела с 18-фтордезоксиглюкозой, однако прогрессирование гиперкортизолемии с развитием стероидного психоза, тяжесть состояния пациента обусловили невозможность дальнейшего ожидания проведения данного визуализирующего исследования, в связи с чем было принято решение о срочном выполнении адреналэктомии. По данным гистологического и иммуногистохимического исследований подтверждено наличие ФЕО, кроме того, была выявлена мелкая аденома коры надпочечника, формирование которой, вероятно, имеет вторичный характер на фоне гиперстимуляции АКТГ. Лабораторный и клинический регресс подтвердил эктопическую секрецию кортикотропина удаленной ФЕО.

АКТГ-эктопический синдром — крайне редкое заболевание, ассоциированное с гиперпродукцией кортикотропина опухолями различной локализации, чаще всего раком легких или карциноидом бронхов. В литературе описано около 65 пациентов с АКТГ-секретирующей ФЕО [9][13–17]. Клиническую картину больных с подобными опухолями объединяют жалобы на общую и мышечную слабость, снижение массы тела, повышенный уровень АД. При первоначальном обследовании обычно выявляются гипокалиемия и гипергликемия, при дальнейшем гормональном исследовании — повышенные уровни АКТГ, кортизола и метанефринов в биологических жидкостях, а при визуализирующих исследованиях обнаруживают новообразование надпочечника. После адреналэктомии обычно отмечается снижение уровней данных гормонов [13][14][16][18–20].

В представленном случае АКТГ-продуцирующая ФЕО сочеталась с другими неоплазиями эндокринных органов: макроаденомой гипофиза без убедительных данных за гормональную активность, первичным гиперпаратиреозом с множественным поражением околощитовидных желез, многоузловым нетоксическим зобом. Подобный комплекс опухолей в отсутствие повышенного уровня кальцитонина, с доброкачественным результатом цитологического исследования материалов ТАБ узлов щитовидной железы более характерен для синдрома Вермера. Генетическое тестирование не выявило у пациента мутации MEN1, однако важно учитывать, что выполнено исследование только 2 и 10 экзонов гена, в связи с чем нельзя исключить наличие дефектов других участков гена. Согласно клиническим рекомендациям Endocrine Society, диагностика синдрома МЭН 1 типа может быть основана на сочетании характерных опухолей эндокринной системы даже в отсутствие генетического подтверждения [3].

Данные о встречаемости АКТГ-продуцирующей ФЕО при синдромах МЭН ограничены. Среди 58 пациентов в работе Gabi J.N. и соавт. (2018) только у 4 выполнено генетическое исследование, при этом в 3 случаях мутаций выявлено не было, а у 1 пациента определена клинически не значимая мутация гена VHL [13]. Описаны единичные случаи подобных опухолей при синдроме МЭН 2А типа [21–23]. В доступной литературе на русском и английском языке мы не нашли описания случая кортикотропин (АКТГ)-продуцирующей ФЕО у пациента при синдроме МЭН 1 типа, на основании чего предполагаем, что представленный клинический случай является первым.

## ЗАКЛЮЧЕНИЕ

ФЕО является редкой гормонально-активной опухолью надпочечников. В связи с нейроэндокринной природой опухоли мозгового вещества надпочечника могут обладать эктопической гормональной активностью. В литературе описан ряд подобных случаев с гиперпродукцией кортикотропина, кальцитонина, метэнкефалина, соматостатина, вазоактивного интестинального пептида и других пептидных гормонов. ФЕО в составе синдрома МЭН 1 типа встречается крайне редко, менее чем в 1% случаев. Пациентов с эктопической гиперпродукцией АКТГ феохромоцитомой в составе синдрома МЭН 1 типа, подобных описанному в данной статье, в доступной литературе на русском и английском языках найдено не было, в связи с чем есть основания считать представленный случай первым. Уникальность описанного случая представляет особенный интерес как с научной, так и с клинической точки зрения.

## ДОПОЛНИТЕЛЬНАЯ ИНФОРМАЦИЯ

Источник финансирования. Работа выполнена по инициативе авторов без привлечения финансирования.

Конфликт интересов. Авторы декларируют отсутствие явных и потенциальных конфликтов интересов, связанных с содержанием настоящей статьи.

Участие авторов. Все авторы одобрили финальную версию статьи перед публикацией, выразили согласие нести ответственность за все аспекты работы, подразумевающую надлежащее изучение и решение вопросов, связанных с точностью или добросовестностью любой части работы.

Согласие пациента. Пациент добровольно подписал информированное согласие на публикацию персональной медицинской информации в обезличенной форме в журнале Проблемы эндокринологии.
